# Hypophosphatasia: Current Literature for Pathophysiology, Clinical Manifestations, Diagnosis, and Treatment

**DOI:** 10.7759/cureus.8594

**Published:** 2020-06-13

**Authors:** Abdulai Bangura, Lisa Wright, Thomas Shuler

**Affiliations:** 1 Department of Research, Trinity School of Medicine, Ratho Mill, VCT; 2 Department of Orthopaedics, Carilion Clinic, Roanoke, USA

**Keywords:** hypophosphatasia, tnap protein human, rickets, osteomalacia, asfotase alfa, serum alkaline phosphatase

## Abstract

Hypophosphatasia (HPP) is a rare inherited bone disorder identified by impaired bone mineralization. There are seven subtypes of HPP mainly characterized by their age of onset. These subtypes consist of perinatal (prenatal) benign, perinatal lethal, infantile, childhood, adult, odontohypophosphatasia, and pseudohypophosphatasia. Due to limited awareness of the condition, either misdiagnosis or delayed diagnosis is common. Furthermore, the condition is frequently treated with contraindicated drugs. This literature illustrates the most recent findings on the etiology, pathophysiology, clinical manifestations, diagnosing, and treatment for HPP and its subtypes. The etiology of the disease consists of loss-of-function mutations of the ALPL gene on chromosome one, which encodes for tissue nonspecific isoenzyme of alkaline phosphatase (TNAP).

A decrease of TNAP reduces inorganic phosphate (Pi) for bone mineralization and allows for an increase in inorganic pyrophosphate (PPi) and phosphorylated osteopontin (p-OPN), which further reduces bone mineralization. The combination of these processes softens bone and mediates a clinical presentation similar to rickets/osteomalacia. HPP has an additional wide range of clinical features depending on its subtype.

Although a concrete diagnostic guideline has not yet been established, many studies have supported a similar method of identifying HPP. Clinical features, radiological findings, and/or biomarker levels of the disorder should raise suspicion and encourage the inclusion of HPP as a differential diagnosis. Biomarkers, especially alkaline phosphatase (ALP), are major contributors to diagnosis. However, genetic testing is done for definitive diagnosis.

The primary treatment for HPP is the reintroduction of TNAP as a recombinant enzyme called asfotase alfa. There are additional pharmaceutical treatments and in some cases, surgical intervention may be indicated. Pharmaceutical therapies such as bisphosphonates, denosumab, potent antiresorptive agents, and vitamin D are contraindicated in adults with HPP.

We hope to raise awareness for HPP in order to prevent delayed diagnosis or misdiagnosis. We plan to encourage appropriate care and avoid treatments that may be contraindicating. We also encourage the development of a diagnostic guideline that will promote a consistently favorable patient prognosis.

## Introduction and background

Hypophosphatasia (HPP) is a rare inherited bone disorder characterized by impaired bone mineralization. The documented epidemiologies of the disorder have been incongruent and contradicting to each other thus far. Its exact prevalence remains unknown and can vary depending on population, method of assessment, and HPP subtype. However, the prevalence has been estimated to range from 1/100,000 to 1/900,000 live births and has been estimated to be 1/2,500 live births within a population with an associate founder effect [1]. HPP has a wide range of clinical features depending on its subtypes. These subtypes are mainly characterized by their age of onset [2]. There are seven subtypes consisting of perinatal (prenatal) benign, perinatal lethal, infantile, childhood, adult, odontohypophosphatasia, and pseudohypophosphatasia [3,4]. Clinical features are more severe with earlier onset subtypes with the exception of prenatal benign HPP. Earlier onset subtypes typically have a worse prognosis due to a higher risk for developing lethal complications such as respiratory insufficiency, caused by rib cage abnormalities, and/or seizures [2]. Due to limited awareness of the condition, delayed diagnosis is common and misdiagnosis is prevalent, leading to a worse prognosis [1,5]. Furthermore, the condition is frequently treated with contraindicated drugs [6]. We cover the most recent findings on the etiology, pathophysiology, clinical manifestations, diagnosing, and treatment for HPP and its subtypes.

## Review

Etiology

The etiology of the disease consists of loss-of-function mutations of the ALPL gene on chromosome one, which encodes for tissue nonspecific isoenzyme of alkaline phosphatase (TNAP) [7]. Earlier onset subtypes are more likely to have an autosomal recessive inheritance pattern, while later-onset subtypes may be inherited in either an autosomal recessive or autosomal dominant pattern [8]. TNAP mainly functions in the bone, liver, and kidneys, but can be identified in other tissues as well. Currently, there have been more than 300 different mutations of the ALPL gene identified, all of which can cause a decrease of TNAP activity. This decrease of TNAP activity initiates a sequence of events leading to a bone softening manifestation similar to rickets/osteomalacia [1].

Pathophysiology

In normal/benign skeletal tissue, TNAP hydrolyzes inorganic pyrophosphate (PPi). This reaction generates inorganic phosphate (Pi), a by-product necessary for matrix vesicle (MV)-mediated hydroxyapatite formation (calcium-phosphate crystallization). Pi can also be produced by another enzyme that is within MVs: phosphoethanolamine/phosphocholine phosphatase from the PHOSPHO1 gene [9]. A decrease in Pi results in a decrease of hydroxyapatite formation. Therefore, we can expect a decrease in bone mineralization with a defect in either the ALPL gene or PHOSPHO1 gene. Up to date, there have not been any mutations/disorders identified with the human PHOSPHO1 gene. However, many recent studies have shown reduced bone matrix mineralization, skeletal abnormalities, and spontaneous fractures in knock-out (KO) mice for the PHOSPHO1 gene [10]. 

Apart from Pi generation for hydroxyapatite formation, TNAP functions by way of decreasing PPi and phosphorylated osteopontin (p-OPN), which both serve as inhibitors of bone mineralization in order to control calcification. PPi prevents hydroxyapatite from being laid within the collagenous scaffolding of the bone matrix [11]. Impaired bone calcification has also been presented in TNAP KO mice studies [12]. In addition to this mechanism, nucleotide pyrophosphatase/phosphodiesterase one (NPP1) resides outside of MVs and produces PPi from adenosine five'-triphosphate (ATP). It is important to note that NPP1 may act as a phosphatase and generate Pi in the absence of TNAP in an attempt to maintain bone mineralization [13]. At the same time, phosphorylated osteopontin (p-OPN) directly binds to hydroxyapatite and prevents it from being involved in bone mineralization. In order for bone mineralization to occur, TNAP dephosphorylates p-OPN to OPN and PPi to Pi [10]. Overall, a decrease of TNAP reduces Pi for bone mineralization and allows for an increase of PPi and p-OPN, which further reduces bone mineralization. The combination of these processes softens bone and mediates a clinical presentation similar to rickets/osteomalacia [1]. However, in the case of rickets/osteomalacia, the underlying cause of low hydroxyapatite formation is insufficient calcium from low dietary calcium and vitamin D intake as opposed to reduced Pi [14]. 

Other than impaired bone mineralization, HPP patients are known to experience epilepsy. TNAP is responsible for Vitamin B6 functioning, converting pyridoxal five’-phosphate (PLP) into pyridoxal (PL), a form more capable of crossing the blood-brain barrier. Without this reaction, PL is unable to be utilized for neurotransmitter synthesis in the CNS and HPP patients can experience seizures [15]. Seizures are more likely experienced by HPP patients with earlier-onset subtypes [2].

Clinical manifestations

In addition to bone and neurological manifestations, HPP patients can experience complications related to growth/development and many systems, including muscular, respiratory, and renal. These patients are also known to have a classic presentation of dental abnormalities [16]. Furthermore, HPP patients could experience rheumatologic-like symptoms [17]. The symptoms are highly variable between each patient and some may even be asymptomatic, particularly adult HPP patients [6]. Although the various HPP forms are mainly characterized by their age of onset, subtypes are known to have overlapping signs, symptoms, and complications. Multiple studies have been reviewed in order to compile these manifestations into a table for better differentiation (Table 1) [1,18-22].

**Table 1:** Hypophosphatasia Subtypes and their Clinical Manifestations The table represents the signs, symptoms, or complications that are associated with the subtypes of HPP. We compiled the listed manifestations from multiple studies [1,18-22]. Abbreviation: HPP, hypophosphatasia

Signs, Symptoms, or Complications	Prenatal/Perinatal benign	Perinatal (in utero to <4 weeks)	Infantile (4 weeks to <6 months)	Childhood (≥ 6 months to < 18 years)	Adult (≥18 years)	Odontohypophosphatasia	Pseudohypophosphatasia
Prenatal skeletal defects with spontaneous improvement	✓						
Can present as any subtype but with normal lab values							✓
Only dental phenotype						✓	
Defective skeletal mineralization	✓	✓	✓	✓			
Early loss of teeth		✓	✓	✓	✓	✓	
Rickets/Osteomalacia		✓	✓	✓	✓		
Chest deformities (e.g rachitic chest)		✓	✓	✓			
Fractures/pseudofractures		✓	✓	✓	✓		
Seizures (Vit B6 responsive)		✓	✓				
Failure to thrive		✓	✓	✓			
Respiratory complications		✓	✓				
Craniosynostosis		✓	✓	✓			
Ophthalmic calcifications		✓	✓	✓	✓		
Bone deformities		✓	✓	✓			
Limb shortening		✓	✓				
Low stature				✓			
Delayed growth			✓				
Delayed bone healing				✓	✓		
Myopathy		✓	✓	✓	✓		
Nephrocalcinosis			✓		✓		
Hypercalcemia			✓	✓			
Hypercalciuria			✓				
Chronic muscle/bone pain				✓	✓		
Arthropathy w/wo chondrocalcinosis					✓		
Enthesopathy					✓		
Pseudogout/calcium pyrophosphate deposition/crystal arthropathy					✓		
Altered gait				✓	✓		
Osteochondral spurs		✓	✓				
Hearing loss		✓	✓				
Intracranial hemorrhages		✓	✓				
Stillbirth		✓					
Death		✓	✓				

Diagnosis

Due to the bone and multi-systemic effect of HPP, patients are commonly misdiagnosed in favor of more common bone and rheumatologic diseases [1]. Some of the recognized misdiagnosis are rickets, osteomalacia, osteogenesis imperfecta, osteoporosis, osteoarthritis, periodontal disease, and chondrodysplasia with abnormal bone mineralization [2,16]. Misdiagnosis can worsen the prognosis by way of two factors: delayed and contraindicated treatment. Many drugs that are used for most bone disorders, like bisphosphonates, can worsen the symptoms for HPP patients [1]. Furthermore, one study revealed the commonality of diagnostic delay in HPP patients with a 12 or more months delay in children and approximately 10 years delay in adults. The study concluded that their results reflected a global limited awareness of the disease [5].

Although a concrete diagnostic guideline has not yet been established, many studies have supported a similar method of identifying HPP. Clinical features, radiological findings, and/or biomarker levels of the disorder should raise suspicion and encourage the inclusion of HPP as a differential diagnosis. Biomarkers are a major contributor to diagnosis [6,16,18,23,24]. However, for definitive diagnosis, ALPL gene testing should be done [25]. Two studies attempted to prepare diagnostic guidelines, but these guidelines have not been widely accepted. Both diagnostic guidelines consist of an algorithm with primary and secondary steps of sign/symptom detection and biomarker evaluation, respectively, with gene testing being performed for a definitive diagnosis [2,25].

The different signs, symptoms, or complications involved with each subtype can be viewed in our table, as discussed previously (Table 1). A fetal ultrasonography can be utilized for the early detection of radiological findings in perinatal lethal and perinatal benign HPP. Some of these features include impaired bone mineralization and shortened/deformed long bone. Similar to the signs and symptoms, plain bone X-ray findings are highly variable depending on the age and severity of HPP. X-ray findings may include: impaired bone mineralization, rickets-like changes, bone deformities, bowed long bones, epiphyseal lucencies, osteophytes specifically in the ulna and fibula, craniosynostosis, fractures, and pseudofractures [25]. 

Due to the impairment of TNAP, patients with HPP will have diagnostic biomarkers related to TNAP and its substrates. Since TNAP is an alkaline phosphatase (ALP), patients will have low levels of plasma ALP. It is important to note many conditions reduce plasma ALP levels; therefore, HPP is mainly diagnosed by the exclusion of other diseases [18]. Furthermore, plasma ALP levels change with age. Bone growth is greater in children than in adults; therefore the plasma ALP normal range for children is higher than the normal range for adults [23]. Other than decreased plasma ALP levels, HPP patients may have increased plasma PPi levels, increased plasma PLP levels, and increased urine phosphoethanolamine (PEA) levels [18]. 

Treatment and management

The primary treatment for HPP is enzyme replacement therapy (ERT). HPP patients suffer from the deficiency/absence of TNAP. Therefore the reintroduction of TNAP as a recombinant enzyme called asfotase alfa provides improvement. In fact, perinatal HPP was lethal either at birth or one month until the introduction of asfotase alfa [26]. In some forms of HPP, the signs/symptoms are less severe and may not require enzyme replacement [25]. Additional treatments that were studied and have shown improvement in HPP patients include teriparatide and monoclonal anti-sclerostin antibodies. Teriparatide is a recombinant human parathyroid hormone. It has been shown to improve pain, mobility, and fracture repair in HPP patients [6]. However, it cannot be used in children [24]. Monoclonal anti-sclerostin antibody has increased bone formation and reduced bone resorption in HPP patients. It has also been proven to increase the bone mineral density (BMD) in HPP patients [27]. It is important to note that pharmaceutical therapies such as bisphosphonates, denosumab, and potent antiresorptive agents may have adverse effects in adults with HPP patients. Furthermore, vitamin D supplementation may exacerbate hypercalcemia or hypercalciuria [6]. On the contrary, the restriction of calcium and vitamin D, or the addition of thiazide diuretics could be used to manage hypercalcemia or hypercalciuria. Additional supportive therapies for HPP patients may include ventilatory support, physiotherapy, occupational therapy, chronic pain management, and fracture care. In some cases, non-healing fractures or craniosynostosis can require surgical intervention [23,24]. 

## Conclusions

The current etiology, pathophysiology, clinical manifestations, diagnosing, and treatment for HPP have been established. The pathophysiology outlined the cause for alteration in the biomarkers except for urine PEA levels. The reason for the increased urine PEA level is unknown. We speculate that the metabolism of PEA involves TNAP. Further research should be carried out to determine the influence of TNAP deficiency on urine PEA levels. Through various research studies, we have acquired the necessary information to identify and diagnose patients with HPP. However, there is a commonality of delayed and misdiagnosis. We believe that this is due to the failure to establish a clear diagnostic guideline. Although two studies have suggested diagnostic guidelines, they have not been widely accepted. 

This literature highlights the most recent findings of HPP in order to raise awareness and prevent delayed diagnosis or misdiagnosis. We also aim to encourage appropriate care and avoid treatments that may be contraindicated. Finally, we hope to urge the derivation of a diagnostic guideline to promote a consistently favorable patient prognosis.
